# Cardiometabolic and Microbiome Effects of Spices and Herbs

**DOI:** 10.1093/nutrit/nuaf267

**Published:** 2026-05-26

**Authors:** Penny M Kris-Etherton, Connie J Rogers, Ester S Oh, Sheila G West, Amandeep K Sandhu, Britt Burton-Freeman, Yudai Huang, David N Proctor, Kristina S Petersen

**Affiliations:** Department of Nutritional Sciences, Pennsylvania State University, University Park, PA 16802, United States; Department of Nutritional Sciences, Pennsylvania State University, University Park, PA 16802, United States; Department of Nutritional Sciences, Pennsylvania State University, University Park, PA 16802, United States; Department of Nutritional Sciences, Pennsylvania State University, University Park, PA 16802, United States; Department of Biobehavioral Health, Pennsylvania State University, University Park, PA 16802, United States; Department of Food Science and Nutrition, Illinois Institute of Technology, Chicago, IL 60616, United States; Department of Food Science and Nutrition, Illinois Institute of Technology, Chicago, IL 60616, United States; Department of Food Science and Nutrition, Illinois Institute of Technology, Chicago, IL 60616, United States; Department of Kinesiology, Pennsylvania State University, University Park, PA 16802, United States; Department of Nutritional Sciences, Pennsylvania State University, University Park, PA 16802, United States

**Keywords:** spices and herbs, cardiovascular disease risk factors, blood pressure, postprandial responses, microbiome

## Abstract

This article appears as part of the supplement “The Role of Spices and Herbs on Supporting Healthy Diets and Improving Nutritional Status,” sponsored by the McCormick Science Institute. Studies conducted at Penn State University to evaluate the effects of spices and herbs (S&H) on risk factors for cardiovascular disease (CVD) are summarized herein. We also report effects of S&H on phytochemical metabolites and the microbiome. Results demonstrate beneficial effects of S&H on postprandial endothelial function, insulin and triglycerides, pancreatic lipase, inflammatory markers, and measures of oxidative defense. In a controlled-feeding trial that evaluated 3 doses of S&H (0.5, 3.3, and 6.6 g/d per 2100 kcal) in the context of an average American diet, the high-S&H diet improved 24-hour blood pressure after 4 weeks, the moderate-S&H diet decreased proinflammatory cytokines, and the high-S&H diet reduced monocyte adherence. Our research also identified polyphenol metabolites that may have important functional properties for CVD risk reduction. Finally, we report benefits of S&H on gut bacterial composition, which suggests possible benefits on CVD risk.

## INTRODUCTION

Cardiovascular disease (CVD) is the leading cause of morbidity and mortality globally and in the United States. According to the 2025 Heart Disease and Stroke Statistics from the American Heart Association (AHA), CVD accounted for 19.4 million deaths globally in 2021 and 941 652 deaths in the United States in 2022.^1^ In 2023, CVD accounted for more deaths in the United States than did the other 2 highest disease-related deaths combined (ie, all types of cancer and chronic lower respiratory diseases). Moreover, between 2017 and 2020, 127.9 million US adults (48.6%) had some form of CVD. The economic toll in the United States is staggering; direct and indirect costs of total CVD were $417.9 billion in 2020 and 2021.^1^ The economic burden is expected to increase markedly over the next 3 decades because of many factors, including increases in the prevalence of obesity, diabetes, and uncontrolled blood pressure, especially at younger ages, and increases in both the aging population with CVD/CVD risk factors and individuals from underserved populations who are at higher risk of CVD.^2^ Consequently, prevention is critical to reduce the high and growing burden of CVD.

Accumulating evidence suggests that spices and herbs (S&H) may have cardiovascular benefits. A substantial body of evidence from clinical trials suggests that large supplemental doses of individual S&H may improve lipids and lipoproteins, glycemic control, blood pressure, adiposity, inflammation, and oxidative stress.^3^ To expand the clinical evidence base, we conducted several randomized controlled studies examining the effect of blends of S&H on risk factors for cardiometabolic diseases. We intentionally studied blends of commonly consumed S&H, selected because of their in vitro antioxidant benefits (reviewed by Jiang^4^). We began with 2 preliminary postprandial studies to establish proof of concept. We confirmed that high doses (≥14 g) were well tolerated and that insulin, triglycerides (TG), and antioxidant activity were significantly improved.^5^ This was followed by further analyses to examine outcomes beyond traditional postprandial measurements. This work formed the basis of a larger controlled-feeding study with nested studies of diverse and innovative outcomes. The goal of this controlled-feeding study was to determine if short-term improvements in cardiometabolic risk achieved with high, acute doses are longer-lasting with daily (chronic) consumption at lower culinary doses.

## POSTPRANDIAL STUDIES OF SPICES AND HERBS

### Effects of Spices and Herbs on Postprandial Insulin, Triglycerides, and Antioxidant Response

Because of the antioxidant properties of S&H, we studied postprandial oxidative stress in response to a high-fat meal with and without S&H. We examined whether adding 14 g of a high-antioxidant S&H blend to a high-fat meal (1200 kcal, 45 g fat) impacted markers of plasma antioxidant status and metabolism.^5^ The S&H tested included the following: black pepper, cinnamon, cloves, garlic powder, ginger, oregano, paprika, rosemary, and turmeric. A previous study by Li et al^6^ showed that a similar blend reduced oxidative stress in response to charred beef. Men with overweight or obesity (mean body mass index [BMI] = 36 kg/m^2^; *n* = 6, age 30–65 years) consumed a control meal and a meal with S&H in a randomized crossover design, with 1 week between testing sessions. High-fat meals were consumed 2 hours after a low-antioxidant meal. Blood was collected before the high-fat meal and at 30-minute intervals for 3.5 hours. Mixed linear models demonstrated treatment × time interactions (*P* values < .05) for insulin (-21%) and TG (-31%) for the meal with S&H vs the control meal. Adding S&H to the meal significantly increased the ferric-reducing antioxidant power, such that postprandial increases following the meal with S&H were 2-fold greater than after the control meal (*P* = .009). Plasma hydrophilic oxygen radical absorbance capacity (ORAC) was also increased by S&H (*P* = .02). There were no treatment differences in glucose, total thiols, lipophilic ORAC, or total ORAC. We concluded that the incorporation of a high dose of S&H into a meal reduces postprandial insulin and TG and enhances antioxidant defense in vivo. These findings suggest that S&H are a potent, low-energy modality for improving physiological responses to high-fat meals.

### Effects of Spices and Herbs on Postprandial Lipemia, Lipase Activity in Response to Psychological Stress

The goal of our next study was to confirm these metabolic effects in a larger sample, and to consider the influence of acute psychological stress.^7^ We used in vitro methods to examine whether lipase inhibition could explain lower TG levels after the high-S&H meal. We used a 2 × 2, randomized, 4-period crossover design to compare the effects of 14.5 g of the same S&H blend incorporated into a high-fat meal (1000 kcal, 45 g fat), followed by standardized psychological stress tests (simulated public speaking and mental arithmetic) vs rest. Adults with overweight or obesity (BMI >25 kg/m^2^), but who were otherwise healthy (*n* = 20; aged 30–65 years and including 6 women), were recruited. Some participants had moderate hypertension. During the control meal, participants were told that the placebo capsules contained antioxidants to address blinding. Blood and systemic hemodynamics were sampled at baseline and at 105, 140, 180, and 210 minutes postmeal. Additional in vitro analyses examined the effect of the S&H blend and constituent S&H on the activity of pancreatic lipase (PL) and secreted phospholipase A_2_ (PLA_2_). Mixed models were used to model the effects of S&H and stress and their interaction. Serum TG, glucose, and insulin were elevated following the meal (*P* < .01). The S&H reduced postmeal TG by 31% when the meal was followed by the rest condition (*P* = .048), but this effect was not present during stress. There was no effect of the S&H blend on glucose or insulin; however, as expected, acute stress significantly increased both measures (*P* < .01; mean increase of 47% and 19%, respectively). The S&H blend and several of the individual S&H dose-dependently inhibited PL and PLA_2_ activity in vitro. The S&H had no effect on blood pressure or systemic vascular resistance at rest or during stress. Surprisingly, the S&H blend increased the heart rate response to stress. In summary, inclusion of S&H may attenuate postprandial lipemia via inhibition of PL and PLA_2_. However, psychological stress exposure negates any influence of the S&H blend on TG, perhaps because the fight-or-flight response shunts blood away from the splanchnic circulation and reduces hepatic clearance. Stress and S&H interacted to increase heart rate.

### Postprandial Effects of Spices and Herbs on Endothelial Function and Lipemia

The purpose of this study was to evaluate the postprandial effects of lower doses of S&H that could be realistically included in a diet typically consumed by the general population.^8^ We were also interested in evaluating the postprandial effects of S&H consumption in a mixed meal on flow-mediated dilation (FMD), which we did not evaluate previously. This pilot study was important to inform the doses of S&H to study in the clinical trial we subsequently conducted to evaluate the effects of S&H on cardiometabolic risk factors. This was a 3-period randomized, controlled, crossover study in which participants (13 men, 52 ± 9 years who had overweight or obesity and an enlarged waist circumference, 102 ± 9 cm) consumed 0 g, 2 g, and 6 g of mixed S&H (basil, bay leaf, black pepper, cinnamon, coriander, cumin, ginger, oregano, parsley, red pepper, rosemary, thyme, turmeric) in a high-saturated-fat, high-carbohydrate meal (1076 kcal, 39 g saturated fat, 98 g carbohydrate). Blood was drawn at baseline and hourly for 4 hours, and FMD was measured at 2 and 4 hours. The main finding was that the meal with 6 g of S&H lessened the postprandial reduction in FMD compared with the meal with no S&H (−0.87% ± 0.32%; *P* = .031). Triglyceride levels were lower after the meal with 2 g of S&H vs after the no-S&H meal (−18 ± 6 mg/dL; *P* = .015). The importance of this study was that it provided evidence that lower doses of S&H (2 g and 6 g) than previously tested attenuated the negative effects of a high-fat, high-carbohydrate meal on lipids and endothelial function. Importantly, beneficial effects were observed with amounts of S&H that could reasonably be attained in an average American diet.

### Postprandial Effects of Spices and Herbs on Inflammation

Many studies have highlighted the anti-inflammatory effects of individual S&H and their bioactive compounds. The effects of an S&H blend, as commonly incorporated into meals, on inflammatory mediators have not been systematically examined in humans in a randomized controlled trial. Accordingly, in this 3-period randomized, controlled, crossover study, we aimed to examine the effect of consuming an S&H blend in a standardized high-saturated-fat, high-carbohydrate test meal compared with the same meal without the S&H blend, on postprandial inflammation in 12 middle-aged men with overweight/obesity.^9^ The current findings were secondary outcome analyses from the clinical trial described above.^8^ Blood samples were collected before and hourly for 4 hours after the meal consumption. The percentage of CD14^+^/human leukocyte antigen–DR isotype^+^ monocytes, as well as proinflammatory cytokine concentrations in plasma and lipopolysaccharide (LPS)-stimulated peripheral blood mononuclear cell (PBMC) culture supernatants were quantified. There was a meal-by-time interaction on interleukin (IL)-1β (*P* < .001), IL-8 (*P* = .020), and tumor necrosis factor alpha (TNF-α) (*P* = .009) secretion from LPS-stimulated PBMCs. IL-1β secretion from LPS-stimulated PBMCs was reduced 4 hours after the meal containing 6 g of S&H, but not 2 g, compared with the 0-g S&H meal. These findings suggest that S&H may have dose-dependent anti-inflammatory potential, and that incorporating them in daily meals may help lower postprandial inflammation and concurrently alleviate chronic low-grade inflammation.

## CONTROLLED-FEEDING STUDY OF SPICES AND HERBS AND CVD RISK

### Effects of Chronic Feeding of Spices and Herbs on CVD Risk

Our previous research showed that a single meal containing a mixture of S&H attenuated postprandial lipemia, reduced proinflammatory cytokine secretion and oxidative stress, and lessened the postprandial reduction in endothelial function.^5^^,^^7–9^ However, previous studues had not examined the longer-term effects of S&H on cardiometabolic risk factors at doses that could realistically be achieved in an average American diet (AAD). Thus, we sought to determine the longer-term effects of S&H in amounts that could typically be consumed in an AAD on risk factors for cardiometabolic diseases.^10^ Because we were interested in the independent effects of an S&H blend, we studied different doses in the context of an AAD (and not a heart-healthy diet). Our clinical trial evaluated the effects of 3 doses of S&H: 0.5 g/day per 2100 kcal (low-spice diet [LSD]), 3.3 g/day per 2100 kcal (moderate-spice diet [MSD]), and 6.6 g/day per 2100 kcal (high-spice diet [HSD]) on lipids and lipoproteins, other risk factors for cardiometabolic diseases. A 3-period, randomized, crossover, controlled-feeding study with 71 male and female participants (30–75 years of age) who were at risk for cardiometabolic diseases (based on elevated BMI and risk factors for metabolic syndrome) was conducted. Each experimental diet was consumed for 4 weeks with a minimum 2-week washout period. Outcomes (lipids, lipoproteins, insulin, glucose, high-sensitivity C-reactive protein [hs-CRP], vascular health measurements including central and peripheral blood pressure and augmentation index, carotid-femoral pulse-wave velocity, ambulatory blood pressure, and flow-mediated dilation, as well as measures of proinflammatory cytokines) were assessed at baseline and at the end of each diet period. Our main finding was that the addition of a relatively high culinary dosage of mixed S&H (6.6 g/d/2100 kcal) improved 24-hour blood pressure after 4 weeks, compared with lower dosages (0.5 and 3.3 g/d/2100 kcal), in the context of an AAD. Specifically, we reported between-diet differences for mean 24-hour systolic (*P* = .02) and diastolic (*P* = .005) ambulatory blood pressure. The HSD lowered mean 24-hour systolic blood pressure compared with the MSD (-1.9 mm Hg; 95% CI, -3.6, -0.2 mm Hg; *P* = .02); the difference between the HSD and LSD was not statistically significant (-1.6 mm Hg; 95% CI, -3.3, 0.04 mm Hg; *P* = .058). The HSD lowered mean 24-hour diastolic blood pressure compared with the LSD (-1.5 mm Hg; 95% CI, -2.5, -0.4 mm Hg; *P* = .003). There were no between-diet effects for clinic-measured blood pressure, low-density-lipoprotein (LDL) cholesterol, markers of glycemia, or vascular function. The importance of this study is that the addition of S&H to an AAD lowered 24-hour systolic and diastolic blood pressure in at-risk adults by a margin that has been shown to confer clinical benefits.^11^

### Effects of Spices and Herbs on Inflammation and Monocyte Function

While numerous studies highlight the acute anti-inflammatory effects of individual S&H, including our previous study demonstrating a postprandial anti-inflammatory effect of consuming an S&H blend delivered in a high-saturated-fat, high-carbohydrate meal,^9^ the effects of longer-term consumption of S&H remain unexplored. Thus, in this study, we aimed to examine the longer-term effect of consuming S&H as part of an AAD on inflammatory mediators related to cardiometabolic risk.^12^ The current findings were secondary outcomes of a 3-period, randomized, crossover, controlled-feeding study including 71 middle-aged men and women at cardiometabolic risk, and the detailed study design was previously described.^10^ At baseline and after each 4-week intervention, proinflammatory cytokines in plasma and LPS-stimulated PBMC culture supernatants were measured. Our main findings showed a reduction in IL-6 levels in plasma and LPS-stimulated PMBC culture supernatants following 4 weeks of the MSD, but not the LSD or HSD. The greater anti-inflammatory effect of the MSD compared with HSD may indicate a nonlinear relation, potentially a U-shaped dose–response, between S&H consumption and the regulation of inflammatory mediators. The HSD may contain a high phenolic content with pro-oxidant properties, potentially leading to oxidative stress by either generating reactive oxygen species or inhibiting the antioxidant defense system.^13^ To explore an alternative aspect of how S&H consumption influences anti-inflammatory effects, we also assessed the phenotype and function of monocyte subsets. Monocyte infiltration into the arterial wall plays a key role in atherogenesis and accelerates disease progression. Each monocyte subset (ie, classical, intermediate, and nonclassical) is characterized by unique phenotypic and functional properties that regulate inflammation and may contribute to atherogenesis.^14^ Classical monocytes have the greatest ability for trans-endothelial migration, intermediate monocytes display robust proinflammatory responses, and nonclassical monocytes patrol the vascular endothelium and detect damaged cells. The consumption of an HSD reduced the percentage of circulating classical monocytes, and the MSD reduced the percentage of circulating intermediate monocytes. Moreover, the HSD reduced the adherence of classical monocytes and the migration of intermediate and nonclassical monocytes. Collectively, these findings suggest that consuming S&H can alter the phenotype and function of monocytes, which may, in part, contribute to the anti-inflammatory effects of S&H consumption and further reduce the risk of atherosclerosis. The key takeaway from this study is that even modest inclusion (3.3 and 6.6 g/d/2100 kcal) of S&H as part of an AAD can exhibit an anti-inflammatory effect.

### Characterization of Phytochemical Metabolites After Consumption of Spices and Herbs

Spices and herbs contain phytochemical compounds, including flavonoids, alkaloids, terpenoids, and phenolic acids, with potential health benefits. These and other phytochemical compounds are transformed through the gastrointestinal tract environment and by host and microbial metabolism, generating molecules available for local and systemic bioactivity. Limited information is available on the metabolites of S&H intake, especially after regular intake, and in conjunction with health outcomes. Accordingly, we analyzed fasting blood samples using ultra-high-performance liquid chromatography coupled with triple quadrupole mass spectrometry from participants (*n* = 63; those who completed all 3 diet periods) at risk of CVD^15^ from the study by Petersen et al.^10^ Preliminary analysis was conducted with representative samples (pooled fasting blood samples from randomly selected individuals; *n* = 10) after consuming the highest dose (6.6 g/d/2100 kcal) of S&H in the controlled diets for 4 weeks. A total of 91 metabolites were identified by mass spectral and fragmentation patterns compared against reference databases, literature, and/or standards. Seventy-one phenolic acids were quantified, including parent and conjugated forms of benzoic acids (612 ± 16 nmol/L), benzaldehydes (92 ± 3 nmol/L), cinnamic acids (40 ± 5 nmol/L), phenylpropionic acids (311 ± 11 nmol/L), phenylacetic acids (123 ± 3 nmol/L), and hippuric acids (2808 ± 35 nmol/L). Eleven terpenoids, including their glucuronide conjugated forms, were quantified (545 ± 48 nmol/L), along with 3 glucuronide conjugated flavonoids (6 ± 0.7 nmol/L), 3 sulfur-containing compounds (496 ± 110 nmol/L), and 3 other compounds (0.23 ± 0.05 nmol/L capsaicin glucuronide isomer, 68 ± 4 nmol/L hydroxycoumarin isomer, 41 ± 9 nmol/L piperine). These data demonstrate that phytochemicals in S&H are bioavailable, appearing in systemic circulation as products of host and microbial metabolism, the latter maximizing yield and diversity of metabolites from phytochemically dense S&H, increasing their potential for imparting health benefits when consumed. Additionally, knowledge of the metabolites generated from intake of foods and ingredients may serve as biomarkers of intake. Select phytochemicals only present in specific S&H can be used in this way, such as piperine in black pepper and *S*-allylcysteine in onion and garlic, detected as the parent form in blood—likewise, capsaicin in red pepper and chili powder; carnosic acid, carnosol, and rosmanol in rosemary; coumarin in cinnamon; and gingerols in ginger. Importantly however, is an understanding of their absorption and clearance kinetics, as glucuronide conjugates of coumarin and gingerols were absent in the representative pooled samples, possibly due to their elimination kinetics, as suggested by our prior work where coumarin- and gingerol-glucuronides peaked after 2 hours and had returned to baseline (nearly undetectable) concentrations by 24 hours of intake.^16^ In contrast, carnosic acid, carnosol, and rosmanol glucuronide conjugates are elevated 24 hours after intake; hence, they were detected at quantifiable concentrations (170 ± 16, 176 ± 11, and 2.7 ± 0.2 nmol/L, respectively) in the current study after an overnight fast. Some phenolic acid metabolite patterns can also reveal the intake of specific phytochemicals, whereas others are nonspecific. The metabolite concentrations quantified in the pooled sample are consistent with prior research. The next steps for this research include quantification of S&H metabolites in blood samples collected from each participant at all dosage levels (0.5, 3.3, and 6.6 g/d), followed by analyses to better understand the relationship between S&H phytochemical metabolism and clinical outcomes, such as endothelial function and blood pressure. These findings may help to develop more effective S&H supplementation strategies, enhancing cardiovascular health and optimizing individual responses.

### Effects of Spices and Herbs on the Microbiome

There has been limited investigation of S&H on the microbiome. A pilot-randomized, placebo-controlled study showed that daily intake of a 5-g capsule containing S&H (cinnamon: 1 g [20%]; oregano: 1.5 g [30%]; ginger: 1.5 g [30%]; black pepper: 0.85 g [17%]; cayenne pepper: 0.15 g [3%]) resulted in a difference in 26 operational taxonomic units (OTUs) when compared with a placebo (maltodextrin) after 2 weeks.^18^ The purpose of this study was to examine gut bacterial composition following an AAD (carbohydrate: 50% kcal; protein: 17%; total fat: 33%; saturated fat: 11%) containing a wider range of S&H at 0.5, 3.3, and 6.6 g/day per 2100 kcal (LSD, MSD, and HSD, respectively) in adults at risk of CVD.^17^ This was a prespecified exploratory analysis conducted using fecal samples collected during the parent study described earlier.^10^ Briefly, fecal samples collected at baseline and following each of the 3 test diets were analyzed using 16S rRNA sequencing. We observed a tendency towards higher alpha diversity, assessed by Faith’s phylogenetic diversity metric, following the MSD vs the LSD. No other between-diet differences in alpha diversity, assessed by Pielou’s evenness or observed features, or beta diversity were observed. At the taxonomic level, between-diet differences in the abundance of several taxa were observed. Notably, the Ruminococcaceae family was enriched after the HSD as compared with the MSD and LSD. We also saw enrichment of the *Agathobacter* genus (a known short-chain fatty acid producer) after the HSD, compared with the MSD and LSD. These findings suggest that culinary doses of S&H may modulate gut bacterial composition in a way that may confer health benefits within 4 weeks in adults at increased risk of CVD.

## SUMMARY

The research summarized herein demonstrates the benefits of S&H on several CVD risk factors. Notably, there are benefits on vascular health—postprandially, TG and insulin clearance are enhanced and oxidative stress is reduced, while longer-term ambulatory blood pressure is decreased. In addition, there are benefits of S&H on markers of inflammation, oxidative stress, and immune function. The studies showing effects on polyphenol metabolites and the microbiome are suggestive of further health benefits conferred by S&H. Further studies are needed to confirm the results we have reported and to identify the optimal dose of individual S&H and S&H blends in various cohorts to elicit the maximum reduction in CVD risk. Longer-term studies are needed with different study participants (ie, those who have hypertension and are of different races and ethnicities) and study conditions that vary relative to S&H dose and the background diet, as well as participant blinding.

These findings suggest that S&H favorably modulate some of the underlying mechanisms that contribute to the development of CVD. Foremost among these longer-term outcomes is the significant decrease in blood pressure. In 2019, according to the Global Burden of Disease (GBD) study, the leading causes of death were ischemic heart disease, stroke, chronic obstructive pulmonary disease, and lower respiratory infections.^19^ High systolic blood pressure was the number 1 metabolic risk factor, and kidney dysfunction (linked in large part to high blood pressure) was number 5, with dietary risks being the number 1 behavioral risk factor for CVD.^20^ Collectively, our findings suggest that dietary doses of S&H may improve blood pressure and decrease CVD risk.
